# Risk of type 2 diabetes according to traditional and emerging anthropometric indices in Spain, a Mediterranean country with high prevalence of obesity: results from a large-scale prospective cohort study

**DOI:** 10.1186/1472-6823-13-7

**Published:** 2013-02-06

**Authors:** José María Huerta, María-José Tormo, María-Dolores Chirlaque, Diana Gavrila, Pilar Amiano, Larraitz Arriola, Eva Ardanaz, Laudina Rodríguez, María-José Sánchez, Michelle Mendez, Diego Salmerón, Aurelio Barricarte, Rosana Burgui, Miren Dorronsoro, Nerea Larrañaga, Esther Molina-Montes, Conchi Moreno-Iribas, José Ramón Quirós, Estefanía Toledo, Noémie Travier, Carlos A González, Carmen Navarro

**Affiliations:** 1 Department of Epidemiology, Murcia Regional Health Authority, Murcia, Spain; 2 CIBER Epidemiología y Salud Pública (CIBERESP), Madrid, Spain; 3 Department of Sociosanitary Sciences, University of Murcia School of Medicine, Murcia, Spain; 4 Public Health Department of Gipuzkoa, Basque Government, San Sebastián, Spain; 5 Public Health Institute of Navarra, Pamplona, Spain; 6 Public Health Directorate, Asturias, Spain; 7 Andalusian School of Public Health, Granada, Spain; 8 Center for Environmental Epidemiology Research, Barcelona, Spain; 9 Autonomous University of Barcelona, Barcelona, Spain; 10 University of Navarra, Pamplona, Spain; 11 Unit of Nutrition, Environment and Cancer, Cancer Epidemiology Research Programme, Catalan Institute of Oncology (ICO), Barcelona, Spain

**Keywords:** Diabetes, Anthropometry, Obesity, Abdominal obesity, Body mass index, EPIC, Spain

## Abstract

**Background:**

Obesity is a major risk factor for type 2 diabetes mellitus (T2DM). A proper anthropometric characterisation of T2DM risk is essential for disease prevention and clinical risk assessement.

**Methods:**

Longitudinal study in 37 733 participants (63% women) of the Spanish EPIC (European Prospective Investigation into Cancer and Nutrition) cohort without prevalent diabetes. Detailed questionnaire information was collected at baseline and anthropometric data gathered following standard procedures. A total of 2513 verified incident T2DM cases occurred after 12.1 years of mean follow-up. Multivariable Cox regression was used to calculate hazard ratios of T2DM by levels of anthropometric variables.

**Results:**

Overall and central obesity were independently associated with T2DM risk. BMI showed the strongest association with T2DM in men whereas waist-related indices were stronger independent predictors in women. Waist-to-height ratio revealed the largest area under the ROC curve in men and women, with optimal cut-offs at 0.60 and 0.58, respectively. The most discriminative waist circumference (WC) cut-off values were 99.4 cm in men and 90.4 cm in women. Absolute risk of T2DM was higher in men than women for any combination of age, BMI and WC categories, and remained low in normal-waist women. The population risk of T2DM attributable to obesity was 17% in men and 31% in women.

**Conclusions:**

Diabetes risk was associated with higher overall and central obesity indices even at normal BMI and WC values. The measurement of waist circumference in the clinical setting is strongly recommended for the evaluation of future T2DM risk in women.

## Background

Obesity is recognised as a major cause of type 2 diabetes mellitus (T2DM) [1-4]. Changing lifestyles over the last decades have given rise to a global epidemic of overweight and obesity which has spread from developed to developing countries and from adults to children and adolescents [5-7]. In 2000, 15% of the Spain population were obese [8], and 9% suffered from T2DM [9]. If trends remain unchanged, conservative estimates are that 12% of the country population will be diabetic by 2030 [9]. The associated future burden of chronic diseases and health costs of the obesity epidemic are of serious concern.

Obesity is defined by the accumulation of excess body fat with potential harmful health effects [10]. A strong link between excess body weight and T2DM risk has long been established in the epidemiological literature. Further evidence showed that ectopic visceral fat accumulation, but not subcutaneous adipose tissue [11], largely accounted for the metabolic complications of obesity [12], such as abnormalities in glucose and lipid metabolism [13] and hepatic insulin resistance [13,14], preceding the development of T2DM.

Anthropometry provides the universal basis for the clinical identification of obese people because the anthropometric methodology is easy to implement, inexpensive, and valid. Simple measures such as height, weight or waist and hip circumferences characterise overall and regional adiposity near as accurately as sophisticated reference methods [11]. The body mass index (BMI), which reflects body general adiposity, is valid for defining obesity at the population level but does not properly account for the wide variation in body fat distribution within individuals [10,15]. BMI does not differentiate between fat and muscle mass [15], follows a non-linear association with body fat percent [16] and is poorly prognostic of obesity-related co-morbidities in subjects of short stature [17] or older age [18]. A proper characterisation of obesity-associated risk requieres that at least one indicator of visceral fat depots is measured. Waist measures such as waist circumference (WC) or waist-to-hip ratio (WHR) are stronger proxies for abdominal obesity than body mass index [11,19]. Even for narrow ranges of BMI large differences in WC exist that account for sizeable variations in risk of chronic conditions such as diabetes or cardiovascular disease [12,20], suggesting that indices of general and central adiposity provide complementary information. On an individual basis, there is discrepancy about which measure would better predict risk of T2DM as two previous meta-analyses found no clear differences for BMI, WC or WHR as predictors of diabetes [21,22]. A further recent meta-analysis suggested that waist-to-height ratio (WHtR) would show a superior predictive ability than BMI or WC, although of limited clinical utility [23]. Using a combination of anthropometric measures to account for both the amount and distribution of body fat seems the best way of characterising obesity-associated T2DM risk [1,2]. Body proportions related to height, such as the WHtR, relative height or leg length, cause further variations in BMI which are not associated with body fat percent [24,25], and may provide additional insight into the association of obesity and T2DM [1,26-28].

Given the aetiologic effect of excess body fat on T2DM and in order for preventive actions to be effective, the most accurate tools for the early identification of at-risk subjects must be adopted. No specific cut-offs exist yet for defining central obesity in Spanish population on the basis of WC and there is little prospective evidence in support of the appropriateness of applying American [29] or European [30] standards to the country population. The main objective of the present study was to obtain specific estimates of diabetes risk in Spain according to different anthropometric variables in a large cohort of participants from the European Prospective Investigation into Cancer and Nutrition (EPIC)-Spain study, and to define those anthropometric values that would better predict future risk of T2DM in this population.

## Methods

### Study sample

The EPIC (European Prospective Investigation into Cancer and Nutrition) Study is an ongoing multi-centre prospective cohort study on diet, genetic and environmental factors and health. The study cohort involves over half a million participants from 10 European countries. The Spanish branch of EPIC comprises 41 438 participants mostly 30–65 years old at the time of enrolment (1992–1995). Participants were recruited mainly among blood donnors but also civil servants and general population from five Spanish regions, three in the North (Asturias, Gipuzkoa and Navarra) and two in the South (Granada and Murcia). Baseline data collection included the measure of anthropometric variables and questionnaire information on diet, lifestyles, medical history and drug consumption, as detailed elsewhere [31,32].

All participants voluntarily agreed to take part in the study and gave informed consent. Ethical approval was granted by the Medical Ethical Committee of Bellvitge Hospital.

### Assessment of anthropometric variables

Participants were invited to attend a physical examination in order to obtain relevant anthropometric information, according to standardised procedures. Height was registered by having the subjects barefoot and in upright position. With participants seated, sitting height was defined as the length from the seat to the top of the head, and leg length was computed as height minus sitting height. The sitting height ratio was then calculated as sitting-height divided by standing height. Weight was assessed with subjects in light underwear using a digital scale with a precision of 0.1 kg. Waist circumference was measured at the narrowest torso circumference for most participants, but the midpoint between the lower ribs and the iliac crest was used instead if the natural waist could not be identified. Finally, hip circumference was registered at the widest diameter of the buttocks. Height, and waist and hip circumferences were measured to the nearest 1 cm.

Body mass index was obtained as weight (in kg) divided by square height (in m). Waist-to-hip and waist-to-height ratios were computed as the quotient between waist and hip circumferences and between WC and height (in cm), respectively.

Full anthropometric data was available for 98.4% of participants.

### Identification of incident diabetes cases

A total of 2560 verified incident T2DM cases occurred between recruitment and December 31st, 2006 (mean follow-up time of 12.1 years). A sensitive approach was used for the ascertainment of T2DM cases based upon different sources of information including self-reported diabetes or consumption of diabetes medication during a follow-up interview 3 years after recruitment, hospital discharge databases, drug prescription records, and regional mortality registers and the National Death Index, for all centres, and record linkage with primary care registers for all centres except Granada (where access to primary care data was partly available for case verification, however). Furthermore, access to laboratory data on glycaemia and glycosilated hemoglobin (HbA_1c_) tests was available in Gipuzkoa.

Verification of possible T2DM cases was carried out by a team of trained health professionals by careful revision of clinical data and health information from all available sources. A definite case was defined if a physician diagnosis of type 2 diabetes was present in the medical history or otherwise evidence of diabetes from two independent sources (depending on study centre), according to the following criteria, 2-hour post-load glycaemia value ≥ 200 mg/dl after a 75-g oral glucose tolerance test (OGTT), HbA_1c_ > 7%, fasting plasma glucose ≥ 126 mg/dl, non-fasting glycaemia ≥ 200 mg/dl, diabetes related medical visit (code E11._ of the 10th revision of the *International Classification of Diseases* (ICD-10)), self-report of diabetes, use of antidiabetic medication (A10 code of the World Health Organization Anatomical Therapeutic Chemical classification system [33]), or death certificate with ICD-10 code E11.

Incidence date was defined either as the earliest date of diagnosis or first antidiabetic drug use registered in the medical records, or the date of self-report. If only information on the month or year was available, the date of diabetes onset was defined at the middle of the corresponding period. For self-reported cases, when several self-reports were available, the incidence date was imputed at the midpoint between the date of the first positive report of diabetes by the participant and the previous negative self-report. Time at risk was calculated as the difference between age at T2DM diagnosis, death, or lost to follow-up, whichever occurred first, and age at recruitment. The ascertainment and verification process of T2DM cases followed the criteria and procedures defined in the EPIC-InterAct study. Further details can be found elsewhere [34].

Exclusions affected prevalent T2DM cases (n = 2383), participants with missing follow-up data on diabetes status (n = 713) and four non-type 2 diabetics. Participants with implausible anthropometric values (height < 130 cm; BMI < 16 kg/m^2^; WC > 160 cm; or WC < 60 cm and BMI > 25 kg/m^2^) (n = 6), or missing data on anthropometry (n = 599) were further excluded. Finally, 14 019 men and 23 714 women completing 2513 incident cases of diabetes and near 457 000 person-years were available for analysis.

### Covariate assessment

Information on habitual diet of the previous year was gathered by means of a validated dietary history method during a personnal interview. Correction for mis-reporting of energy inake was applied by classifying participants as under-reporters, plausible reporters and over-reporters according to the predicted total energy expenditure (pTEE) method, as described by Méndez *et al.*[35]. A daily consumption of alcohol between 10–50 g in men and 5–25 g in women was regarded as ‘moderate’, with ‘low’ and ‘high’ consumption groups defined outside these ranges. Information was also collected on smoking status (never, former, current, unknown), educational level (primary school or lower, secondary or technical/professional school, university, unknown), and practice of recreational activities. Total MET-hours/week in walking, cycling and sports were computed to derive a four-category recreational physical activity index: inactive, moderately inactive, moderately active, active.

### Statistical analyses

Descriptive statistics by sex and diabetes status included median and interquartile range for continuous variables, and absolute and relative frequencies for categorical ones. Mann–Whitney U tests or *χ*^2^ test were applied to evaluate differences in the distribution of the data by diabetes status, as appropriate. Analyses were performed separately for men and women.

Hazard ratios of diabetes by sex-specific quartiles of anthropometric variables were computed for men and women separately taking the lowest quartile as the reference. Cox proportional hazards models were built with attained age as the time variable (entry time defined as age at recruitment and exit time defined as age at incident diabetes or age at censoring). Cox models were stratified by centre, age (in five-year categories), and follow-up time (<5 years, 5–10 years, ≥10 years). The validity of the proportional hazards assumption was tested on the basis of Schoenfeld residuals. All analyses were controlled for total energy intake, plausibility of energy reporting, alcohol intake level, smoking status, educational attainment, recreational physical activity index, and menopausal status, in women. In order to analyse the independency of the reported associations, BMI models were further adjusted for WC, and central obesity models were adjusted for height and weight. Other covariates such as dietary (consumption of proteins, lipids, carbohydrates, red meat, processed meat, tea, coffee, fiber, and magnesium), or parity variables (in women), had no significant influence on risk estimates and were not included in the final models. Furthermore, several sensitivity analyses were conducted in order to test the robustness of results by excluding participants with short follow-up or chronic conditions at baseline, or by stratifying the results by categories of age, follow-up time and menopausal status.

The area under the receiver operating characteristic (ROC) curve was estimated for selected anthropometric variables and optimal cutoffs were defined at the maximum Youden index (J = sensitivity + specificity – 1). Sensitivity and specificity values were also computed for WC at the National Cholesterol Education Program-Adult Treatment Panel III (NCEP-ATPIII) and International Diabetes Federation (IDF) cutoffs.

The 10-year cumulative incidence of diabetes was calculated according to NCEP-ATPIII waist categories, stratified by BMI (normal weight, overweight, obese), sex, and age group (40–49, 50–59, 60–69 years), as the cumulative number of cases at 10 years of follow-up divided by the total population in each stratum.

The population risk of diabetes attributable to excess body weight was calculated as *p*_*d*_*(RR - 1)/RR*; where *p*_*d*_ = prevalence of exposition among cases, and *RR* = multivariable-adjusted hazard ratio of diabetes in exposed *versus* non-exposed participants [36].

Non-linear associations of diabetes risk with BMI and WC were also modelled using restricted cubic splines with sex-specific distribution-based knots at the 5th, 25th, 50th, 75th and 95th percentiles of BMI and WC. Relative hazards of diabetes derived from adjusted Cox models were then plotted against continuous variables. Nelson-Aalen cumulative hazard estimates by BMI and WC categories were plotted separately for men and women.

Analyses were performed using STATA 10.1 (StataCorp LP, College Station, USA). All tests were two-sided and *P*-values < 0.05 were considered significant.

## Results

As shown in Table 1, the incidence of diabetes was higher in men than women, not showing clear geographical patterns. Cases were, in general, older, fatter, less educated, and less physically active (Table 2). In addition, diabetic men were more prone to smoke, as opposed to diabetic women. Overall, differences in anthropometric variables between cases and non-cases were larger in women.

**Table 1:** Incidence rates of diabetes in the Spanish EPIC (European Prospective Investigation into Cancer and Nutrition) cohort, by sex and center

	**Men**				**Women**			
	**Person-years**	**Cases**	**Crude rate**	**Truncated standardised rate**^**1**^	**Person-years**	**Cases**	**Crude rate**	**Truncated standardised rate**^**1**^
Total	170499	1278	750	605 (565–645)	290047	1252	432	407 (380–433)
Asturias	35031	258	737	613 (512–714)	64689	266	411	399 (345–453)
Gipuzkoa	45155	345	764	567 (507–628)	50635	195	385	355 (304–405)
Navarra	43894	342	779	572 (511–634)	48810	239	490	433 (378–488)
Granada	18572	99	533	421 (334–507)	62464	245	392	354 (310–399)
Murcia	27847	234	840	725 (321–829)	63450	307	307	433 (378–488)

Rates per 100 000 person-years (with 95% confidence intervals) for the 30–64 age band.

^1^ Adjusted to the European Standard Population using the direct method.

**Table 2:** Baseline general and anthropometrical characteristics of diabetic and non-diabetic members of the Spanish EPIC (European Prospective Investigation into Cancer and Nutrition) cohort, by sex

	**Men**				**Women**			
	**Non-cases**		**Cases**		**Non-cases**		**Cases**	
	**Median**	**IQR**	**Median**	**IQR**	**Median**	**IQR**	**Median**	**IQR**
Age (years)	49.3	11.6	52.2	11.4	47.1	13.3	51.9	12.1
Height (cm)	169.0	8.4	168.0	7.9	157.0	7.8	155.2	8.0
Weight (kg)	80.0	13.4	84.5	15.4	66.8	13.8	76.4	16.8
Waist circumference (cm)	98.0	11.0	103.5	12.0	85.0	15.0	97.0	14.0
Hip circumference (cm)	104.0	7.7	106.6	9.0	104.0	11.0	111.0	14.0
Sitting height (cm)	87.3	5.3	86.8	4.9	82.7	4.7	82.2	4.8
Leg length (cm)	81.8	6.3	81.0	6.3	74.2	5.8	73.0	5.6
Body mass index (kg/m^2^)	27.9	4.1	30.0	4.5	27.1	5.9	31.8	6.5
Waist-to-hip ratio	0.94	0.06	0.97	0.06	0.82	0.08	0.86	0.07
Waist-to-height ratio	0.58	0.07	0.62	0.07	0.54	0.10	0.63	0.09
	**N**	**%**	**N**	**%**	**N**	**%**	**N**	**%**
BMI categories^1^								
Normal weight	1876	14.7	68	5.3	6637	29.5	63	5.1
Overweight	7572	59.4	569	44.7	9658	43.0	379	30.5
Obese	3299	25.9	635	49.9	6178	27.5	799	64.4
WC categories^2^								
Low	3619	28.4	132	10.4	6582	29.3	48	3.9
Medium	4813	37.8	409	32.2	6501	28.9	175	14.1
High	4315	33.9	731	57.5	9390	41.8	1018	82.0
Educational level								
Primary or lower	7599	59.6	924	72.6	16524	73.5	1032	83.2
Secondary	2748	21.6	225	17.7	2631	11.7	62	5.0
University	2026	15.9	76	6.0	2260	10.1	26	2.1
Unknown	374	2.9	47	3.7	1058	4.7	121	9.8
Smoking								
Never smoker	3864	30.3	290	22.8	15699	69.9	1012	81.5
Former smoker	3861	30.3	360	28.3	2340	10.4	71	5.7
Smoker	5016	39.4	620	48.7	4422	19.7	158	12.7
Unknown	6	0.1	2	0.2	12	0.1	0	0
Recreational physical activity index^3^								
Inactive	4353	34.1	472	37.1	8728	38.8	531	42.8
Moderately inactive	3241	25.4	350	27.5	7447	33.1	387	31.2
Moderately active	2765	21.7	258	20.3	4351	19.4	222	17.9
Active	2388	18.7	192	15.1	1947	8.7	101	8.1
Menopausal status								
Pre-menopausal	-		-		12530	55.8	449	36.2
Peri-menopausal	-		-		2109	9.4	141	11.4
Post-menopausal	-		-		7834	34.9	651	52.5

*BMI*: body mass index, *WC*: waist circumference.

All within-sex comparisons between cases and non-cases were significant at *P* < 0.001 level, except for the recreational physical activity index (*P*_*men*_ = 0.003; *P*_*women*_ = 0.051).

^1^ Normal weight, BMI < 25 kg/m^2^; overweight, 25 ≤ BMI < 30 kg/m^2^; obese, BMI ≥ 30 kg/m^2^.

^2^ Low, WC < 94 cm (men) or WC < 80 cm (women); medium, 94 ≤ WC < 102 cm (men) or 80 ≤ WC < 88 cm (women); high, WC ≥ 102 cm (men) or WC ≥ 88 cm (women).

^3^ Sum of MET-h/week spent in walking, cycling and sports. Inactive, ≤19.50; moderately inactive, 19.51-33.75; moderately active, 33.76-54.75; active, >54.75.

Table 3 shows the main results of the sex-specific analyses of diabetes risk by quartiles of anthropometric indices of general (BMI), gluteo-femoral (hip circumference), and central obesity (WC, WHR, or WHtR). The estimated risk of diabetes was generally higher for any level increase of body mass index or waist-related variables. Point estimates of T2DM risk (Q_4_*vs.* Q_1_) ranged from 1.5 for hip circumference to 2.6 for BMI, in men, and from 2.5 for hip circumference to 7.9 for WHtR, in women. Adjustment of BMI models for indices of central obesity did not affect the estimation of diabetes risk in men, but led to an attenuation of risk estimates in women. All variables remained independent predictors of T2DM risk in mutually adjusted models, except hip circumference.

**Table 3:** Hazard ratio (HR) of diabetes by quartiles of anthropometrical indices in men and women from the Spanish EPIC (European Prospective Investigation into Cancer and Nutrition) cohort

	**Men**						**Women**					
	**Py**	**Cases**	**HR**^**1**^	**95%CI**	**HR**^**2**^	**95% CI**	**Py**	**Cases**	**HR**^**1**^	**95% CI**	**HR**^**2**^	**95% CI**
Body mass index												
Q1	43152	135	1		1		73322	52	1		1	
Q2	43115	211	1.57	1.27 - 1.97	1.58	1.25 - 2.00	73311	132	1.51	1.08 - 2.11	1.26	0.90 - 1.77
Q3	42155	343	2.02	1.63 - 2.50	2.07	1.64 - 2.61	71690	331	2.89	2.12 - 3.95	2.11	1.52 - 2.93
Q4	40657	583	2.57	2.08 - 3.16	2.68	2.05 - 3.50	69564	726	4.14	3.04 - 5.64	2.48	1.73 - 3.57
Waist circumference												
Q1	43948	118	1		1		79543	43	1		1	
Q2	42483	230	1.58	1.25 - 1.99	1.49	1.18 - 1.90	72348	137	2.19	1.52 - 3.14	2.02	1.40 - 2.91
Q3	42502	353	1.92	1.54 - 2.40	1.74	1.37 - 2.22	69726	320	4.02	2.85 - 5.68	3.47	2.44 - 4.95
Q4	40146	571	2.32	1.86 - 2.86	1.93	1.46 - 2.55	66272	741	5.93	4.21 - 8.36	4.57	3.12 - 6.68
Hip circumference												
Q1	42690	197	1		1		75803	100	1		1	
Q2	44831	273	1.13	0.93 - 1.38	1.00	0.82 - 1.23	71293	192	1.56	1.21 - 2.02	1.24	0.96 - 1.61
Q3	40094	317	1.49	1.23 - 1.80	1.21	0.98 - 1.49	74551	335	1.98	1.56 - 2.52	1.24	0.96 - 1.60
Q4	41464	485	1.49	1.24 - 1.79	0.99	0.78 - 1.27	66241	614	2.50	1.97 - 3.17	1.00	0.73 - 1.35
Waist-to-hip ratio												
Q1	49640	170	1		1		79106	80	1		1	
Q2	38989	226	1.28	1.04 - 1.57	1.17	0.95 - 1.45	77254	208	1.59	1.21 - 2.10	1.50	1.14 - 1.98
Q3	47330	450	1.65	1.37 - 2.00	1.48	1.22 - 1.80	69546	337	2.27	1.74 - 2.97	1.98	1.52 - 2.60
Q4	33121	426	1.63	1.34 - 1.99	1.30	1.05 - 1.60	61983	616	3.18	2.46 - 4.12	2.67	2.06 - 3.47
Waist-to-height ratio												
Q1	43815	105	1		1		73889	30	1		1	
Q2	43545	224	1.53	1.20 - 1.96	1.48	1.15 - 1.89	73835	132	2.91	1.92 - 4.41	2.79	1.76 - 4.06
Q3	41515	365	2.05	1.62 - 2.59	1.91	1.50 - 2.42	71746	314	5.00	3.34 - 7.49	4.54	2.79 - 6.40
Q4	40204	578	2.32	1.84 - 2.93	2.07	1.60 - 2.69	68418	765	7.91	5.30 - 11.82	6.80	4.45 - 10.39

*Py*: person-years, *HR*: hazard ratio, *CI*: confidence interval.

^1^ Models stratified by centre, age, and follow-up time, and adjusted by total energy intake, plausibility of energy reporting, alcohol intake, smoking status, educational level and recreational physical activity.

^2^ Multivariable models mutually adjusted by height, weight, and waist and hip circumferences, as applicable. Covariates, total energy intake, plausibility of energy reporting, alcohol intake, smoking status, educational level, physical activity, and menopausal status (women). Models were stratified by centre, age, and follow-up time.

Cut-off points,

Body mass index (kg/m^2^), 26.12 (Q1-Q2), 28.10 (Q2-Q3), 30.34 (Q3-Q4) [men]; 24.64 (Q1-Q2), 27.37 (Q2-Q3), 30.74 (Q3-Q4) [women].

Waist circumference (cm), 93.0 (Q1-Q2), 98.8 (Q2-Q3), 104.5 (Q3-Q4) [men]; 79.0 (Q1-Q2), 86.0 (Q2-Q3), 94.0 (Q3-Q4) [women].

Hip circumference (cm), 100.5 (Q1-Q2), 104.5 (Q2-Q3), 108.8 (Q3-Q4) [men]; 99.0 (Q1-Q2), 104.5 (Q2-Q3), 111.0 (Q3-Q4) [women].

Waist-to-hip ratio, 0.91 (Q1-Q2), 0.94 (Q2-Q3), 0.98 (Q3-Q4) [men]; 0.78 (Q1-Q2), 0.82 (Q2-Q3), 0.86 (Q3-Q4) [women].

Waist-to-height ratio, 0.55 (Q1-Q2), 0.58 (Q2-Q3), 0.62 (Q3-Q4) [men]; 0.50 (Q1-Q2), 0.55 (Q2-Q3), 0.60 (Q3-Q4) [women].

The areas under the ROC curves for the principal anthropometric variables considered are shown in Table 4. On an individual basis, WHtR revealed the highest area for both sexes, with optimal sensitivity/specificity cut-offs at 0.60 in men and 0.58 in women, as defined by the Youden’s J statistic. Table 5 shows the comparison of discriminatory ability of several anthropometric cut-offs in regard to T2DM. Although more sensitive, the IDF criterion showed poorer specificity (<30%), whereas the NCEP criterion resulted in more balanced sensitivity and specificity estimates and classified participants according to their diabetes status twice as good as the IDF values. In turn, a 0.5 WHtR threshold resulted in very poor specificity in this study.

**Table 4:** Area under the curve (AUC) for different anthropometrical variables as predictors of incident diabetes in men and women from the Spanish EPIC (European Prospective Investigation into Cancer and Nutrition) cohort

	**AUC**	**SE**	**95% CI**	**Optimal cutoff**^**1**^	**Sensitivity**	**Specificity**
MEN						
Body mass index (kg/m^2^)	0.676	0.008	0.660 - 0.691	28.7	66.7%	59.9%
Waist circumference (cm)	0.672	0.008	0.657 - 0.687	99.4	69.5%	55.8%
Waist-to-hip ratio	0.646	0.008	0.631 - 0.661	0.95	68.9%	53.2%
Waist-to-height ratio	0.687	0.008	0.673 - 0.702	0.60	66.1%	61.2%
WOMEN						
Body mass index (kg/m^2^)	0.759	0.006	0.746 - 0.771	29.2	71.8%	66.9%
Waist circumference (cm)	0.773	0.006	0.760 - 0.785	90.4	74.5%	67.6%
Waist-to-hip ratio	0.722	0.007	0.708 - 0.735	0.84	71.0%	61.9%
Waist-to-height ratio	0.776	0.006	0.764 - 0.788	0.58	76.6%	65.6%

*SE*: standard error, *CI*: confidence interval.

^1^ Maximum Youden index (J) value (J = sensitivity + specificity - 1).

**Table 5:** Comparison of predefined anthropometric cut-offs for predicting incident diabetes in men and women from the Spanish EPIC (European Prospective Investigation into Cancer and Nutrition) cohort

	**Sensitivity**	**Specificity**	**Correctly classified**^**1**^
MEN			
Body mass index ≥ 25 kg/m^2^	94.7%	14.7%	22.0%
Body mass index ≥ 30 kg/m^2^	49.9%	74.1%	71.9%
IDF waist circumference (WC ≥ 94 cm)	89.6%	28.4%	34.0%
NCEP waist circumference (WC ≥ 102 cm)	57.5%	66.2%	65.4%
Waist-to-hip ratio ≥ 0.90	94.4%	16.8%	23.8%
Waist-to-height ratio ≥ 0.5	98.8%	4.8%	13.4%
WOMEN			
Body mass index ≥ 25 kg/m^2^	94.2%	29.5%	33.0%
Body mass index ≥ 30 kg/m^2^	64.4%	72.5%	72.1%
IDF waist circumference (WC ≥ 80 cm)	96.1%	29.3%	32.8%
NCEP waist circumference (WC ≥ 88 cm)	82.0%	58.2%	59.5%
Waist-to-hip ratio ≥ 0.85	64.4%	68.4%	68.2%
Waist-to-height ratio ≥ 0.5	97.6%	26.3%	30.1%

*NCEP*: national cholesterol education program, *IDF*: international diabetes federation.

^1^ Proportion of participants with correctly classified diabetes status according to the respective cut-off point of each binary anthropometric variable considered.

In supplementary analyses, height, but not sitting-height ratio, was shown to be significantly associated with a decreased risk of T2DM both in men and women, regardless of total body weight or waist circumference (Additional file 1: Table S1). Predicted T2DM risk was consistently higher at incresing BMI WHO categories, and also independently in those with central obesity according to the NCEP-ATPIII criterion (Additional file 1: Table S2). Results did not change after further adjustment for dietary variables or exclusion of participants with chronic conditions at baseline (Additional file 1: Table S3). However, evidence of heterogeneity existed by follow-up strata, and by groups of age and menopausal status. The effect of a larger WC on T2DM risk was evaluated separately in normal weight, overweight and obese participants (Additional file 1: Table S4). Women presented higher risk estimates at high WC values, with obese participants of large WC (≥ 88 cm) showing up to 5.6-fold times higher risk of diabetes than their normal weight, low WC (< 80 cm) counterparts (*versus* 2.8 times higher risk in men). Of note, less than 5% of women with normal WC became diabetic after 10 years of follow-up, even if obese and in the 60–69 years old group (Additional file 1: Table S5). Absolute risks were higher in men than women for any age, waist and BMI category. The population risk of diabetes attributable to excess body weight (BMI ≥ 25 kg/m^2^) in the EPIC-Spain cohort was estimated in 46% (95% CI, 33 - 59%) for men and 61% (95% CI, 52 - 71%) for women (Additional file 1: Table S6). If only obesity were targeted, the proportion of avoidable cases would reach an estimated 17% (95% CI, 13 - 21%) in men and 31% (95% CI, 27 - 36%) in women.

**Figure 1:**
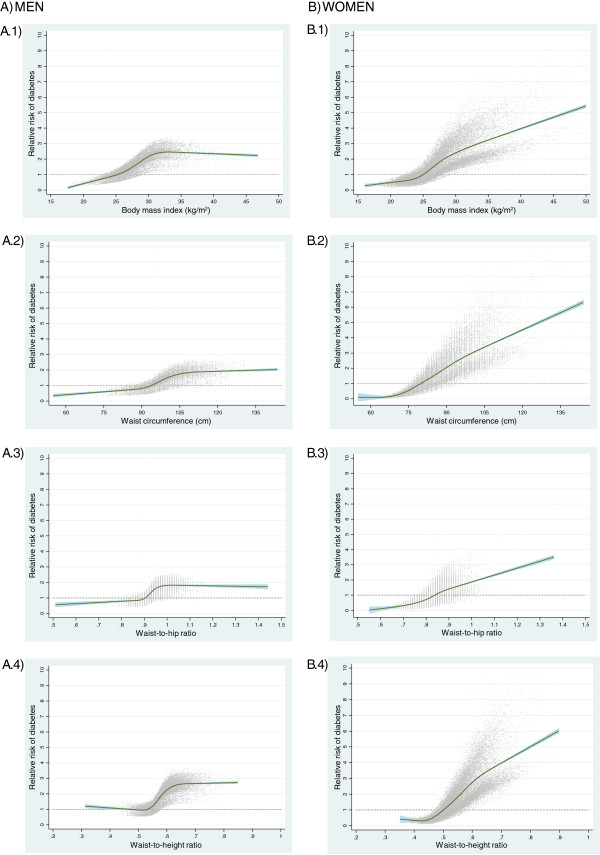
**Relative risk of diabetes as a function of different anthropometric indices in men and women from the Spanish EPIC (European Prospective Investigation into Cancer and Nutrition) cohort.** Restricted cubic splines modelling of T2DM risk according to variation in anthropometric variables, with knots at the 5th, 25th, 50th, 75th, and 95th percentiles. The reference (RR = 1, dashed line) was set at a body mass index equal to 25 kg/m^2^ (**A.1** and **B.1**), a waist circumference equal to 94 cm in men (**A.2**) or 80 cm in women (**B.2**), a waist-to-hip ratio equal to 0.90 in men (**A.3**) or 0.85 in women (**B.3**), and a waist-to-height ratio equal to 0.50 (**A.4** and **B.4**).

The modelling of T2DM risk related to the anthropometric indices evaluated using restricted cubic splines revealed a similar pattern for the studied indices within each sex, but appreciably different between sexes, showing curvilinear relationships with steeper slopes in women, while associations tended to reach a plateau at highest anthropometric values in men (Figure 1).

**Figure 2:**
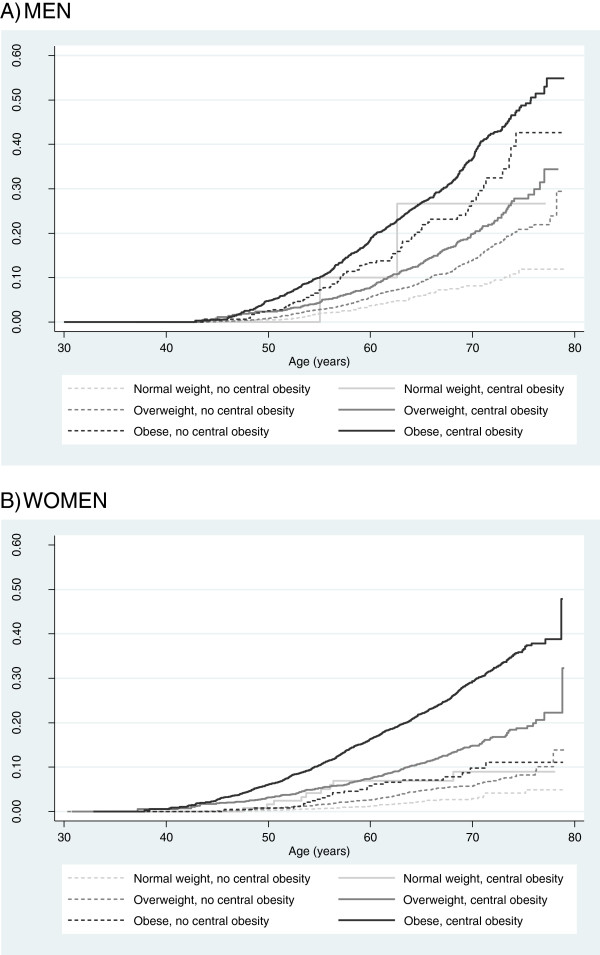
**Nelson-Aalen cumulative hazard estimates of diabetes by combined strata of body mass index and waist circumference in men (A) and women (B) from the Spanish EPIC (European Prospective Investigation into Cancer and Nutrition) cohort.** Body mass index strata (normal weight, overweight, obese) were defined according to WHO standard body mass index groups (< 25, 25-29.99, ≥ 30 kg/m^2^); waist circumference strata were based on the presence of central obesity, defined by a waist circumference ≥ 102 cm in men or a waist circumference ≥ 88 cm in women.

Figure 2 shows the cumulative hazard of diabetes by age for combined BMI and WC strata. Central obesity added independently to the risk of diabetes in each BMI category, but much more significantly in women, for whom the estimated cumulative hazard of diabetes in normal-waist participants remained below 15% even among the oldest.

## Discussion

General and central obesity were both independent predictors of T2DM risk in this large prospective cohort of volunteers from different Spanish regions. Risk estimates at elevated values of BMI and WC were higher for women than for men. Also central obesity, although a moderate independent predictor of T2DM in men, showed the strongest association with diabetes in women. The measure of waist circumference in the clinical practice would thus be a valuable and inexpensive aid in the evaluation of diabetes risk, especially for women.

The practical need to distinguish between general and central obesity from an anthropometric perspective, raises the question about which indicator would be the best proxy for central obesity either in clinical and epidemiological settings. Literature showed that relative measures of WC, such as the waist-to-hip ratio, predicted risk of disease no better than WC alone [22], which guided the definition of central obesity to rely exclusively on WC [29,30]. But recent findings have given support to the use of WHtR as a better central obesity index in the prediction of T2DM among different ethnic groups [26,28,37-39]. Height influences the shape and frame size of individuals, and the evidence suggests that taller people tend to be leaner (and meager) than their shorter counterparts [24]. In the present study, taller participants had around 25% reduced risk of becoming diabetic independently of their weight and abdominal perimeter. Since height is also conceived as a proxy indicator of childhood nutritional status, its strong association with diabetes may thus integrate a double dimension of actual physical characteristics and infancy socio-economic circumstances [40]. Our results supports waist-to-height ratio as the best single measure to predict risk of diabetes, presenting the largest area under the ROC curve in both sexes (AUC_men_ = 0.69; AUC_women_ = 0.78), in line with previous evidence, with an optimal cut-off value of 0.6, above the proposed threshold of 0.5 [37]. The consistency of the predictive ability of WHtR with regard to T2DM across different ethnic backgrounds, and sex groups, together with its simplicity, supports including this promising index systematically in future epidemiological studies in the field of diabetes and other chronic diseases [37,41]. However, the definition of universal clinical thresholds (if at all possible) still warrants further investigation in different age groups and disease contexts. From a clinical point of view, although waist measures provide no dramatic gain in discriminatory ability as compared to BMI, given their independent and graded association with T2DM, the authors firmly believe that it deserves consideration the inclusion of central obesity measures (WC the simplest, WHtR the most discriminative) in clinical practice guidelines for the management of T2DM patients to assist diagnosis and decision-making by the physician, at least in the case of female patients. The combination of BMI and WC would allow a much more accurate ranking of individuals according to their disease risk. Our results show large differences in 10-year absolute risk of T2DM between normal-waist and high-waist female participants, further supporting the relevance of WC as a complementary measure for evaluating diabetes risk in women.

As a point for discussion, no specific cut-off points have yet been adopted to define central obesity in Spain. With regard to diabetes, our results suggest optimal cut-offs at 99 cm in men and 90 cm in women. Two other criteria for defining central obesity are available which are mostly used in the diagnosis of metabolic syndrome, those proposed by the IDF for European population (94/80 cm) [30] and those of the NCEP-ATPIII (102/88 cm) [29]. Although less sensitive, the NCEP-ATPIII criterion was much more specific when applied to our cohort; besides, the IDF criterion failed to correctly classify a large proportion of the sample. Until adapted, specific values are defined, our data strongly supports the adoption of NCEP-ATPIII values in the Spanish population to enable international comparability of country data.

Obesity, both general and central, had a greater influence on the risk of T2DM in women than men, consistent with previous evidence [2]. Genetic effects determine sex differences in body composition [42] and hormonal factors [43] have been invoked to account for the weaker association between obesity and T2DM in men. However, reasons for this sex-specific effect are not clear. A gynoid fat pattern, characterised by fat tissue depots in thighs and hip, has been shown to be inversely associated to fasting and postload glucose concentrations, and to diabetes risk, independently of BMI and waist circumference [44]. Larger thigh and hip circumferences in women could reflect increased femoral and gluteal subuctaneous fat mass, which have been reported to show high lipoprotein lipase activity and low lipolytic activity [44,45], thus contributing to fatty acid uptake and storage [45]. In turn, abdominal fat depots are more strongly related to insulin resistance than periferal or gluteal adiposity by releasing larger amounts of free fatty acids into the blood that become lipotoxic for hepatic and muscle cells [11,46]. Thus, women with an android fat pattern (a pattern of central adiposity) might be at higher relative risk of T2DM because of both a diabetogenic effect of intra-abdominal fat and a reduced ‘protection’ by hip and thigh fat depots, as compared to gynoid females. In support of the latter, Cameron et al. have recently shown the important confounding effect of hip circumference in the association between central obesity and all-cause and cardiovascular mortality, hip circumference being inversely associated to mortality after adjustment by waist circumference [47]. In our study, hip circumference was no longer associated to diabetes risk once weight and waist circumference were accounted for in categorical models, but it showed a significant independent inverse relationship with diabetes in a continuous model (HR_men, per standard deviation_ = 0.90, 95% CI, 0.82 - 0.99; HR_women, per standard deviation_ = 0.73, 95% CI, 0.65 - 0.83; data not shown), which points out to a protective independent effect of hip circumference similar to that suggested by previous authors [44,47]. These results highlight the aetiological role of central obesity in promoting T2DM, but also revive the discussion on the importance of hip circumference as an independent predictor of chronic disease and mortality, especially among women.

The large and increasing prevalence of overweight and obesity, and the ageing of the population in Spain, as in many other countries [6,7,48], raises serious concerns about the future burden of diabetes morbidity and mortality. In our study, we have estimated that around 46% of cases among men, and 61% of cases among women, could be avoided by maintaining a normal weight (BMI < 25 kg/m^2^). If our relative risk estimates were extrapolated to the general Spanish population, using the most accurate and up-to-date nation-wide figures available for Spain (an overall prevalence of overweight or obesity of 70.8% and 53.9% for men and women respectively [49]), the proportion of diabetes cases that could be avoided if the population kept a normal weight could be calculated in 40.1% (95% CI, 27.3 - 52.9%) in men and 49.7% in women (95% CI, 38.9 - 60.4%). Even further gains are conceivable if lower reductions in BMI or abdominal girth were achieved. However, further extrapolation of these results must be applied with caution, since prevalences and relative risk estimates may vary in other settings.

Some limitations should be considered when interpreting these data. The EPIC-Spain sample was not representative of the general population, consisted of a large proportion of blood donors and included predominantly women, which would limit the generalizability of the results. Furthermore, the prevalence of elevated BMI in this cohort was very high as compared to other populations, particularly those from Asian origin [50], this meaning that the estimated risks (relative and aboslute) and cut-off values obtained should not be directly extrapolated to populations of different geographical, ethnic, or cultural contexts. Unfortunately, no data was available on family history of diabetes, and thus the genetic background of participants could not be accounted for in the analyses. The limited availability of primary care data in one of the study centres might not have resulted in a significant misclassification bias, however, since the associations remained unchanged after excluding the participants from this centre in a sensitivity analysis. Finally, since no additional anthropometric measures were performed during the follow-up, the possibility that participants could have lost or put on weight after recruitment could not be evaluated. Furthermore, it was not possible to assess to what extent potential reductions in weight or WC during the study period would be able to decrease T2DM risk in this population. Important strengths are the large sample size, the prospective design with a long follow-up time, and the use of anthropometric measures, not self-reports. Also, the large number of cases available allowed for robust estimates of T2DM risk across the full range of relevant anthropometric indicators. Finally, a large set of confounders was available to control for, including dietary and lifestyle variables, and mis-reporting of energy intake.

## Conclusion

Diabetes risk was consistently associated with higher overall and central obesity indices in adult population, even at BMI and WC values regarded as normal. The study provides specific estimates of diabetes risk (absolute and relative) by categories of anthropometric variables in Spanish men and women, makes the importance of central adiposity indices manifest, especially for women, and promotes the measurement of waist circumference in the clinical setting to assist the evaluation of metabolic risk.

## Abbreviations

BMI: Body mass index; EPIC: European prospective investigation into cancer and nutrition; ICD: International classification of diseases; IDF: International diabetes federation; MET: Metabolic equivalent; NCEP-ATPIII: National cholesterol education program-adult treatment panel III; pTEE: Predicted total energy expenditure; ROC: Receiver operating characteristic; T2DM: Type 2 diabetes mellitus; WC: Waist circumference; WHtR: Waist-to-height ratio; WHR: Waist-to-hip ratio.

## Competing interests

The authors declare that they have no competing interests.

## Authors’ contributions

CN, MJT, MDC, EA, MJS, AB, MD, JRQ and CG participated in the design of the EPIC-Spain study. JMH and DS performed the statistical analyses. JMH, MJT, MDC and CN drafted the manuscript. MDC, DG, PA, LA, EA, LR, MJS, MM, AB, RB, MD, NL, EMM, CMI, ET and NT were involved in data collection and/or made important intellectual contributions to the interpretation of data and the writing of the paper. All authors critically revised and approved the final version.

## Pre-publication history

The pre-publication history for this paper can be accessed here:

http://www.biomedcentral.com/1472-6823/13/7/prepub

## Supplementary Material

Additional file 1 Table S1 Hazard ratio (HR) of diabetes by quartiles of components of height in men and women from the Spanish EPIC (European Prospective Investigation into Cancer and Nutrition) cohort. **Table S2.** Hazard ratio (HR) of diabetes by standard categories of body mass index (BMI) and waist circumference (WC) in men and women from the Spanish EPIC (European Prospective Investigation into Cancer and Nutrition) cohort. **Table S3.** Sensitivity analyses for the association of indicators of general and central obesity and risk of diabetes in men and women from the Spanish EPIC (European Prospective Investigation into Cancer and Nutrition) cohort. **Table S4.** Hazard ratio (HR) and cumulative proportion of diabetes by combined BMI and WC categories in men and women from the Spanish EPIC (European Prospective Investigation into Cancer and Nutrition) cohort. **Table S5.** Ten-years cumulative incidence (%) of diabetes (and 95% CI) according to waist circumference NCEP categories, stratified by body mass index, sex and age, in participants from the Spanish EPIC (European Prospective Investigation into Cancer and Nutrition) cohort. **Table S6.** Population risk of diabetes attributable to excess body weight in men and women from the Spanish EPIC (European Prospective Investigation into Cancer and Nutrition) cohort. Click here for file

## References

[B1] The Diabetes Prevention Program Research Group Relationship of body size and shape to the development of diabetes in the diabetes prevention program Obesity (Silver Spring) 2006 14 11 2107 2117 17135629 10.1038/oby.2006.246 PMC2373982

[B2] Meisinger C Döring A Thorand B Heier M Löwel H Body fat distribution and risk of type 2 diabetes in the general population, are there differences between men and women? The MONICA/KORA Augsburg cohort study Am J Clin Nutr 2006 84 3 483 489 16960160 10.1093/ajcn/84.3.483

[B3] Schienkiewitz A Schulze MB Hoffmann K Kroke A Boeing H Body mass index history and risk of type 2 diabetes, results from the European Prospective Investigation into Cancer and Nutrition (EPIC)-Potsdam Study Am J Clin Nutr 2006 84 2 427 433 16895894 10.1093/ajcn/84.1.427

[B4] Wang Y Rimm EB Stampfer MJ Willett WC Hu FB Comparison of abdominal adiposity and overall obesity in predicting risk of type 2 diabetes among men Am J Clin Nutr 2005 81 3 555 563 15755822 10.1093/ajcn/81.3.555

[B5] Kosti RI Panagiotakos DB The epidemic of obesity in children and adolescents in the world Cent Eur J Public Health 2006 14 4 151 159 17243492 10.21101/cejph.a3398

[B6] Prentice AM The emerging epidemic of obesity in developing countries Int J Epidemiol 2006 35 1 93 99 16326822 10.1093/ije/dyi272

[B7] Rokholm B Baker JL Sorensen TI The levelling off of the obesity epidemic since the year 1999–a review of evidence and perspectives Obes Rev 2010 11 12 835 846 10.1111/j.1467-789X.2010.00810.x 20973911

[B8] Aranceta J Pérez-Rodrigo C Serra-Majem L Ribas-Barba L Quiles-Izquierdo J Vioque J Tur-Marí J Mataix J Llopis J Tojo R Prevalencia de la obesidad en España, resultados del estudio SEEDO 2000 [Prevalence of obesity in Spain, results of the SEEDO 2000 study] Med Clin (Barc) 2003 120 16 608 612 10.1157/13046926 12732125

[B9] Wild S Roglic G Green A Sicree R King H Global prevalence of diabetes, estimates for the year 2000 and projections for 2030 Diabetes Care 2004 27 5 1047 1053 10.2337/diacare.27.5.1047 15111519

[B10] World Health Organization Obesity, preventing and managing the global epidemic. Report of a WHO consultation (2000) 2012 WHO Technical Report Series 894, Geneva 11234459

[B11] Bray GA Jablonski KA Fujimoto WY Barrett-Connor E Haffner S Hanson RL Hill JO Hubbard V Kriska A Stamm E Relation of central adiposity and body mass index to the development of diabetes in the Diabetes Prevention Program Am J Clin Nutr 2008 87 5 1212 1218 18469241 10.1093/ajcn/87.5.1212 PMC2517222

[B12] Després JP Lemieux I Bergeron J Pibarot P Mathieu P Larose E Rodés-Cabau J Bertrand OF Poirier P Abdominal obesity and the metabolic syndrome, contribution to global cardiometabolic risk Arterioscler Thromb Vasc Biol 2008 28 6 1039 1049 10.1161/ATVBAHA.107.159228 18356555

[B13] Gastaldelli A Cusi K Pettiti M Hardies J Miyazaki Y Berria R Buzzigoli E Sironi AM Cersosimo E Ferrannini E Relationship between hepatic/visceral fat and hepatic insulin resistance in nondiabetic and type 2 diabetic subjects Gastroenterology 2007 133 2 496 506 10.1053/j.gastro.2007.04.068 17681171

[B14] Barzilai N She L Liu BQ Vuguin P Cohen P Wang J Rossetti L Surgical removal of visceral fat reverses hepatic insulin resistance Diabetes 1999 48 1 94 98 10.2337/diabetes.48.1.94 9892227

[B15] Stevens J Katz EG Huxley RR Associations between gender, age and waist circumference Eur J Clin Nutr 2010 64 1 6 15 10.1038/ejcn.2009.101 19738633 PMC5909719

[B16] Welch GW Sowers MR The interrelationship between body topology and body composition varies with age among women J Nutr 2000 130 9 2371 2377 10958838 10.1093/jn/130.9.2371

[B17] Lara-Esqueda A Aguilar-Salinas CA Velázquez-Monroy O Gómez-Pérez FJ Rosas-Peralta M Mehta R Tapia-Conyer R The body mass index is a less-sensitive tool for detecting cases with obesity-associated co-morbidities in short stature subjects Int J Obes Relat Metab Disord 2004 28 11 1443 1450 10.1038/sj.ijo.0802705 15356661

[B18] Price GM Uauy R Breeze E Bulpitt CJ Fletcher AE Weight, shape, and mortality risk in older persons, elevated waist-hip ratio, not high body mass index, is associated with a greater risk of death Am J Clin Nutr 2006 84 2 449 460 16895897 10.1093/ajcn/84.1.449

[B19] Chan DC Watts GF Barrett PH Burke V Waist circumference, waist-to-hip ratio and body mass index as predictors of adipose tissue compartments in men QJM 2003 96 6 441 447 10.1093/qjmed/hcg069 12788963

[B20] Onat A Avci GS Barlan MM Uyarel H Uzunlar B Sansoy V Measures of abdominal obesity assessed for visceral adiposity and relation to coronary risk Int J Obes Relat Metab Disord 2004 28 8 1018 1025 10.1038/sj.ijo.0802695 15197408

[B21] Qiao Q Nyamdorj R Is the association of type II diabetes with waist circumference or waist-to-hip ratio stronger than that with body mass index? Eur J Clin Nutr 2010 64 1 30 34 10.1038/ejcn.2009.93 19724291

[B22] Vázquez G Duval S Jacobs DR Jr Silventoinen K Comparison of body mass index, waist circumference, and waist/hip ratio in predicting incident diabetes, a meta-analysis Epidemiol Rev 2007 29 1 115 128 10.1093/epirev/mxm008 17494056

[B23] Kodama S Horikawa C Fujihara K Heianza Y Hirasawa R Yachi Y Sugawara A Tanaka S Shimano H Iida KT Comparisons of the Strength of Associations With Future Type 2 Diabetes Risk Among Anthropometric Obesity Indicators, Including Waist-to-Height Ratio, A Meta-Analysis Am J Epidemiol 2012 176 11 959 969 10.1093/aje/kws172 23144362

[B24] Bogin B Beydoun N The relationship of sitting height ratio to body mass index and fatness in the United States, 1988–1994 Hum Ecol 2012 15 Special Issue S1 S8

[B25] Norgan NG Relative sitting height and the interpretation of the body mass index Ann Hum Biol 1994 21 1 79 82 10.1080/03014469400003092 8147579

[B26] Hadaegh F Zabetian A Harati H Azizi F Waist/height ratio as a better predictor of type 2 diabetes compared to body mass index in Tehranian adult men--a 3.6-year prospective study Exp Clin Endocrinol Diabetes 2006 114 6 310 315 10.1055/s-2006-924123 16868890

[B27] Lawlor DA Ebrahim S Davey SG The association between components of adult height and Type II diabetes and insulin resistance, British Women’s Heart and Health Study Diabetologia 2002 45 8 1097 1106 10.1007/s00125-002-0887-5 12189439

[B28] Shao J Yu L Shen X Li D Wang K Waist-to-height ratio, an optimal predictor for obesity and metabolic syndrome in Chinese adults J Nutr Health Aging 2010 14 9 782 785 10.1007/s12603-010-0106-x 21085910 PMC12880338

[B29] Grundy SM Cleeman JI Daniels SR Donato KA Eckel RH Franklin BA Gordon DJ Krauss RM Savage PJ Smith SC Jr Diagnosis and management of the metabolic syndrome, an American Heart Association/National Heart, Lung, and Blood Institute Scientific Statement Circulation 2005 112 17 2735 2752 10.1161/CIRCULATIONAHA.105.169404 16157765

[B30] Alberti KG Zimmet P Shaw J Metabolic syndrome—a new world-wide definition. A Consensus Statement from the International Diabetes Federation. Diabet Med 2006 23 5 469 480 10.1111/j.1464-5491.2006.01858.x 16681555

[B31] Riboli E Kaaks R The EPIC Project, rationale and study design. European Prospective Investigation into Cancer and Nutrition Int J Epidemiol 1997 26 Suppl 1 S6 S14 9126529 10.1093/ije/26.suppl_1.s6

[B32] Riboli E Hunt KJ Slimani N Ferrari P Norat T Fahey M Charrondiere UR Hemon B Casagrande C Vignat J European Prospective Investigation into Cancer and Nutrition (EPIC), study populations and data collection Public Health Nutr 2002 5 6B 1113 1124 10.1079/PHN2002394 12639222

[B33] Anatomical Therapeutic Chemical Classification Systemhttp://www.whocc.no

[B34] Langenberg C Sharp S Forouhi NG Franks PW Schulze MB Kerrison N Ekelund U Barroso I Panico S Tormo MJ Design and cohort description of the InterAct Project, an examination of the interaction of genetic and lifestyle factors on the incidence of type 2 diabetes in the EPIC Study Diabetologia 2011 54 9 2272 2282 21717116 10.1007/s00125-011-2182-9 PMC4222062

[B35] Mendez MA Popkin BM Buckland G Schroder H Amiano P Barricarte A Huerta JM Quirós JR Sánchez MJ González CA Alternative methods of accounting for underreporting and overreporting when measuring dietary intake-obesity relations Am J Epidemiol 2011 173 4 448 458 10.1093/aje/kwq380 21242302 PMC3139974

[B36] Benichou J Palta M Ahrens W, Pigeot I Rates, Risks, Measures of Association and Impact Handbook of Epidemiology 2005 Springer, Berlin 89 156

[B37] Browning LM Hsieh SD Ashwell M A systematic review of waist-to-height ratio as a screening tool for the prediction of cardiovascular disease and diabetes, 0.5 could be a suitable global boundary value Nutr Res Rev 2010 23 2 247 269 10.1017/S0954422410000144 20819243

[B38] He Y Zhai F Ma G Feskens EJ Zhang J Fu P Van’t VP Yang X Abdominal obesity and the prevalence of diabetes and intermediate hyperglycaemia in Chinese adults Public Health Nutr 2009 12 8 1078 1084 10.1017/S1368980008003856 18986591

[B39] Kondaki K Grammatikaki E Pavon DJ Manios Y Gonzalez-Gross M Sjostrom M Gottrand F Molnar D Moreno LA Kafatos A Comparison of several anthropometric indices with insulin resistance proxy measures among European adolescents, The Helena Study Eur J Pediatr 2011 170 6 731 739 10.1007/s00431-010-1322-4 21052739

[B40] Lawlor DA Davey SG Ebrahim S Life course influences on insulin resistance, findings from the British Women’s Heart and Health Study Diabetes Care 2003 26 1 97 103 10.2337/diacare.26.1.97 12502664

[B41] Hsieh SD Muto T The superiority of waist-to-height ratio as an anthropometric index to evaluate clustering of coronary risk factors among non-obese men and women Prev Med 2005 40 2 216 220 10.1016/j.ypmed.2004.05.025 15533532

[B42] Heid IM Jackson AU Randall JC Winkler TW Qi L Steinthorsdottir V Thorleifsson G Zillikens MC Speliotes EK Magi R Meta-analysis identifies 13 new loci associated with waist-hip ratio and reveals sexual dimorphism in the genetic basis of fat distribution Nat Genet 2010 42 11 949 960 10.1038/ng.685 20935629 PMC3000924

[B43] Haffner SM Sex hormones, obesity, fat distribution, type 2 diabetes and insulin resistance, epidemiological and clinical correlation Int J Obes Relat Metab Disord 2000 24 Suppl 2 S56 S58 10997610 10.1038/sj.ijo.0801279

[B44] Snijder MB Dekker JM Visser M Bouter LM Stehouwer CD Kostense PJ Yudkin JS Heine RJ Nijpels G Seidell JC Associations of hip and thigh circumferences independent of waist circumference with the incidence of type 2 diabetes, the Hoorn Study Am J Clin Nutr 2003 77 5 1192 1197 12716671 10.1093/ajcn/77.5.1192

[B45] Rebuffé-Scrive M Enk L Crona N Lonnroth P Abrahamsson L Smith U Bjorntorp P Fat cell metabolism in different regions in women. Effect of menstrual cycle, pregnancy, and lactation J Clin Invest 1985 75 6 1973 1976 10.1172/JCI111914 4008649 PMC425556

[B46] Després JP Is visceral obesity the cause of the metabolic syndrome? Ann Med 2006 38 1 52 63 10.1080/07853890500383895 16448989

[B47] Cameron AJ Magliano DJ Shaw JE Zimmet PZ Carstensen B Alberti KG Tuomilehto J Barr EL Pauvaday VK Kowlessur S The influence of hip circumference on the relationship between abdominal obesity and mortality Int J Epidemiol 2012 41 2 484 494 10.1093/ije/dyr198 22266094 PMC3324456

[B48] Tzotzas T Krassas GE Prevalence and trends of obesity in children and adults of South Europe Pediatr Endocrinol Rev 2004 1 Suppl 3 448 454 16444173

[B49] Gutiérrez-Fisac JL Guallar-Castrillón P León-Muñoz LM Graciani A Banegas JR Rodríguez-Artalejo F Prevalence of general and abdominal obesity in the adult population of Spain, 2008–2010, the ENRICA study Obes Rev 2012 13 4 388 392 10.1111/j.1467-789X.2011.00964.x 22151906

[B50] World Map of Obesityhttp://www.iaso.org/resources/world-map-obesity

