# Supplementary material for: Risk of type 2 diabetes according to traditional and emerging anthropometric indices in Spain, a Mediterranean country with high prevalence of obesity: results from a large-scale prospective cohort study
Source: BMC Endocr Disord. 2013 Feb 6;13:7. doi: 10.1186/1472-6823-13-7 (PMC3575248; doi:10.1186/1472-6823-13-7)
Supplement: Additional file 1 Table S1 — Hazard ratio (HR) of diabetes by quartiles of components of height in men and women from the Spanish EPIC (European Prospective Investigation into Cancer and Nutrition) cohort. Table S2. Hazard ratio (HR) of diabetes by standard categories of body mass index (BMI) and waist circumference (WC) in men and women from the Spanish EPIC (European Prospective Investigation into Cancer and Nutrition) cohort. Table S3. Sensitivity analyses for the association of indicators of general and central obesity and risk of diabetes in men and women from the Spanish EPIC (European Prospective Investigation into Cancer and Nutrition) cohort. Table S4. Hazard ratio (HR) and cumulative proportion of diabetes by combined BMI and WC categories in men and women from the Spanish EPIC (European Prospective Investigation into Cancer and Nutrition) cohort. Table S5. Ten-years cumulative incidence (%) of diabetes (and 95% CI) according to waist circumference NCEP categories, stratified by body mass index, sex and age, in participants from the Spanish EPIC (European Prospective Investigation into Cancer and Nutrition) cohort. Table S6. Population risk of diabetes attributable to excess body weight in men and women from the Spanish EPIC (European Prospective Investigation into Cancer and Nutrition) cohort. [file 1472-6823-13-7-S1.doc]

**Table S1. Hazard ratio (HR) of diabetes by quartiles of components of height in men and women from the Spanish EPIC (European Prospective Investigation into Cancer and Nutrition) cohort.**

|  | Men | | | |  | Women | | | |
| --- | --- | --- | --- | --- | --- | --- | --- | --- | --- |
|  | Py | Cases | HR^1^ | 95% CI |  | Py | Cases | HR^1^ | 95% CI |
| Height |  |  |  |  |  |  |  |  |  |
| Q1 | 44161 | 394 | 1 |  |  | 75256 | 427 | 1 |  |
| Q2 | 40895 | 344 | 0.95 | 0.81 - 1.11 |  | 67825 | 317 | 0.91 | 0.77 – 1.07 |
| Q3 | 41506 | 294 | 0.88 | 0.74 -1.05 |  | 72753 | 284 | 0.96 | 0.81 – 1.14 |
| Q4 | 42516 | 240 | 0.73 | 0.60 - 0.88 |  | 72054 | 213 | 0.77 | 0.63 – 0.94 |
| Sitting height |  |  |  |  |  |  |  |  |  |
| Q1 | 42553 | 357 | 1 |  |  | 71790 | 371 | 1 |  |
| Q2 | 42634 | 367 | 0.97 | 0.83 - 1.14 |  | 72517 | 316 | 1.10 | 0.94 - 1.30 |
| Q3 | 44930 | 305 | 0.98 | 0.82 - 1.16 |  | 74091 | 309 | 0.90 | 0.75 - 1.07 |
| Q4 | 38961 | 243 | 0.78 | 0.63 - 0.95 |  | 69489 | 245 | 1.00 | 0.82 - 1.23 |
| Leg length |  |  |  |  |  |  |  |  |  |
| Q1 | 41893 | 386 | 1 |  |  | 71172 | 410 | 1 |  |
| Q2 | 42749 | 314 | 1.03 | 0.88 - 1.21 |  | 73188 | 332 | 0.98 | 0.83 - 1.15 |
| Q3 | 44015 | 326 | 0.92 | 0.79 - 1.09 |  | 71225 | 259 | 0.89 | 0.75 -1.06 |
| Q4 | 40472 | 246 | 0.80 | 0.67 - 0.97 |  | 72302 | 240 | 0.84 | 0.70 - 1.01 |
| Sitting height ratio |  |  |  |  |  |  |  |  |  |
| Q1 | 45697 | 327 | 1 |  |  | 74709 | 279 | 1 |  |
| Q2 | 40246 | 301 | 0.97 | 0.82 - 1.16 |  | 74756 | 312 | 1.15 | 0.96 - 1.37 |
| Q3 | 42930 | 318 | 0.97 | 0.81 - 1.16 |  | 71502 | 323 | 1.13 | 0.95 - 1.36 |
| Q4 | 40205 | 326 | 1.05 | 0.87 - 1.26 |  | 66921 | 327 | 1.05 | 0.88 - 1.27 |

Py: person-years. HR: hazard ratio. CI: confidence interval.

^1^ Models stratified by centre, age, and follow-up time, and adjusted for weight, waist circumference, total energy intake, plausibility of energy reporting, alcohol intake, smoking status, educational level, recreational physical activity, and menopausal status (women).

Cut-off points:

Height (cm): 165.0 (Q1-Q2), 169.0 (Q2-Q3), 173.2 (Q3-Q4) [men]; 153.0 (Q1-Q2), 156.8 (Q2-Q3), 160.7 (Q3-Q4) [women].

Sitting height (cm): 84.7 (Q1-Q2), 87.3 (Q2-Q3), 90.0 (Q3-Q4) [men]; 80.2 (Q1-Q2), 82.6 (Q2-Q3), 85.0 (Q3-Q4) [women].

Leg length (cm): 78.6 (Q1-Q2), 81.7 (Q2-Q3), 85.0 (Q3-Q4) [men]; 71.2 (Q1-Q2), 74.1 (Q2-Q3), 77.0 (Q3-Q4) [women].

Sitting height ratio: 0.51 (Q1-Q2), 0.52 (Q2-Q3), 0.53 (Q3-Q4) [men]; 0.52 (Q1-Q2), 0.53 (Q2-Q3), 0.54 (Q3-Q4) [women].

**Table S2. Hazard ratio (HR) of diabetes by standard categories of body mass index (BMI) and waist circumference (WC) in men and women from the Spanish EPIC (European Prospective Investigation into Cancer and Nutrition) cohort.**

|  |  | Py | Cases | HR^1^ | 95% CI |  | HR^2^ | 95% CI |
| --- | --- | --- | --- | --- | --- | --- | --- | --- |
| Men |  |  |  |  |  |  |  |  |
| General obesity |  |  |  |  |  |  |  |  |
| Normal range | 18.50 ≤ BMI < 22.50 | 3969 | 5 | 1 |  |  | 1 |  |
| Normal range | 22.50 ≤ BMI < 25.00 | 19781 | 62 | 3.06 | 1.22 - 7.71 |  | 3.05 | 1.21 - 7.67 |
| Overweight | 25.00 ≤ BMI < 27.50 | 48917 | 197 | 3.45 | 1.40 - 8.49 |  | 3.40 | 1.38 - 8.40 |
| Overweight | 27.50 ≤ BMI < 30.00 | 50552 | 372 | 5.47 | 2.24 - 13.38 |  | 5.34 | 2.16 - 13.22 |
| Obese | 30.00 ≤ BMI < 35.00 | 40725 | 502 | 6.62 | 2.71 - 16.20 |  | 6.39 | 2.55 - 16.03 |
| Obese | ≥ 35.00 | 5087 | 133 | 6.93 | 2.78 - 17.25 |  | 6.54 | 2.47 - 17.33 |
|  |  |  |  |  |  |  |  |  |
| Central obesity |  |  |  |  |  |  |  |  |
| No | WC < 102 | 110068 | 541 | 1 |  |  | 1 |  |
| Yes | WC ≥ 102 | 59011 | 731 | 1.43 | 1.26 - 1.62 |  | 1.13 | 0.97 - 1.31 |
|  |  |  |  |  |  |  |  |  |
| Women |  |  |  |  |  |  |  |  |
| General obesity |  |  |  |  |  |  |  |  |
| Normal range | 18.50 ≤ BMI < 22.50 | 26112 | 10 | 1 |  |  | 1 |  |
| Normal range | 22.50 ≤ BMI < 25.00 | 56362 | 53 | 2.04 | 1.03 - 4.06 |  | 1.67 | 0.84 - 3.31 |
| Overweight | 25.00 ≤ BMI < 27.50 | 67213 | 136 | 3.03 | 1.56 - 5.88 |  | 2.11 | 1.09 - 4.11 |
| Overweight | 27.50 ≤ BMI < 30.00 | 55611 | 243 | 5.08 | 2.63 - 9.80 |  | 3.04 | 1.56 - 5.92 |
| Obese | 30.00 ≤ BMI < 35.00 | 60771 | 476 | 6.52 | 3.39 - 12.55 |  | 3.19 | 1.61 - 6.28 |
| Obese | ≥ 35.00 | 21450 | 323 | 8.76 | 4.52 - 16.99 |  | 3.01 | 1.47 - 6.19 |
|  |  |  |  |  |  |  |  |  |
| Central obesity |  |  |  |  |  |  |  |  |
| No | WC < 88 | 164443 | 223 | 1 |  |  | 1 |  |
| Yes | WC ≥ 88 | 123445 | 1018 | 2.58 | 2.18 - 3.04 |  | 1.95 | 1.61 - 2.35 |

BMI: body mass index (kg/m^2^). WC: waist circumference (cm). Py: person-years. HR: hazard ratio. CI: confidence interval.

^1^ Models stratified by centre, age, and follow-up time, and adjusted by total energy intake, plausibility of energy reporting, alcohol intake, smoking status, educational level, recreational physical activity, and menopausal status (women).

^2^ General obesity models adjusted by waist circumference; central obesity models adjusted by height and weight. Covariates: total energy intake, plausibility of energy reporting, alcohol intake, smoking status, educational level, physical activity, and menopausal status (women). Models were stratified by centre, age, and follow-up time.

**Table S3. Sensitivity analyses for the association of indicators of general and central obesity and risk of diabetes in men and women from the Spanish EPIC (European Prospective Investigation into Cancer and Nutrition) cohort.**

|  | Men | | | | |  | Women | | | | |  |
| --- | --- | --- | --- | --- | --- | --- | --- | --- | --- | --- | --- | --- |
|  | BMI (per s.d) | |  | WC (per s.d.) | |  | BMI (per s.d) | | WC (per s.d.) | | |  |
|  | HR | 95% CI |  | HR | 95% CI |  | HR | 95% CI |  | HR | 95% CI |  |
| Whole cohort^1^ | 1.23 | 1.13 - 1.33 |  | 1.01 | 0.91 - 1.12 |  | 1.04 | 0.94 - 1.15 |  | 1.56 | 1.39 - 1.76 |  |
| COVARIATE ADJUSTMENT |  |  |  |  |  |  |  |  |  | |  |  |
| Further adjustment for other dietary variables^2^ | 1.22 | 1.12 - 1.32 |  | 1.01 | 0.91 - 1.12 |  | 1.04 | 0.94 - 1.15 |  | 1.54 | 1.37 - 1.74 |  |
| EXCLUSIONS |  |  |  |  |  |  |  |  |  | |  |  |
| Excluding participants with prevalent cancer or cardiovascular disease^3^ | 1.24 | 1.14 - 1.35 |  | 1.03 | 0.93 - 1.14 |  | 1.06 | 0.95 - 1.18 |  | 1.55 | 1.37 - 1.75 |  |
| Excluding participants within the first 2 years of follow-up (n = 139) | 1.24 | 1.14 - 1.35 |  | 1.01 | 0.91 - 1.12 |  | 1.07 | 0.96 – 1.18 |  | 1.55 | 1.37 - 1.75 |  |
| Excluding participants following a special diet^4^ (n = 3125) | 1.22 | 1.12 - 1.33 |  | 1.04 | 0.93 - 1.16 |  | 1.06 | 0.95 - 1.19 |  | 1.59 | 1.40 - 1.81 |  |
| STRATIFIED ANALYSES |  |  |  |  |  |  |  |  |  | |  |  |
| By age |  |  |  |  |  |  |  |  |  | |  |  |
| < 55 years | 1.29 | 1.19 - 1.41 |  | 0.88 | 0.79 - 0.98 |  | 1.12 | 0.98 - 1.26 |  | 1.46 | 1.26 -1.69 |  |
| ≥ 55 years | 1.41 | 1.20 - 1.65 |  | 0.85 | 0.70 - 1.03 |  | 1.08 | 0.93 - 1.27 |  | 1.24 | 1.03 -1.49 |  |
| *P* for interaction | 0.06 | |  | 0.08 | |  | 0.38 | | 0.10 | | |  |
| By follow-up time |  |  |  |  |  |  |  |  |  | |  |  |
| < 5 years | 1.06 | 0.79 - 1.44 |  | 1.00 | 0.70 - 1.44 |  | 0.82 | 0.64 - 1.06 |  | 1.77 | 1.27 - 2.45 |  |
| 5-10 years | 1.16 | 1.04 - 1.29 |  | 0.92 | 0.80 - 1.06 |  | 1.05 | 0.91 - 1.22 |  | 1.32 | 1.11 - 1.57 |  |
| ≥ 10 years | 1.43 | 1.27 - 1.61 |  | 1.14 | 0.98 - 1.33 |  | 1.19 | 1.01 - 1.40 |  | 1.82 | 1.51 - 2.19 |  |
| *P* for interaction | < 0.01 | |  | < 0.01 | |  | < 0.01 | |  | < 0.01 | |  |
| By menopausal status |  |  |  |  |  |  |  |  |  | |  |  |
| Pre-menopausal | - |  |  | - |  |  | 1.10 | 0.92 - 1.32 |  | 1.59 | 1.29 - 1.95 |  |
| Peri-menopausal | - |  |  | - |  |  | 1.29 | 0.85 - 1.95 |  | 1.45 | 0.88 - 2.40 |  |
| Post-menopausal | - |  |  | - |  |  | 0.96 | 0.84 - 1.11 |  | 1.59 | 1.35 - 1.88 |  |
| *P* for interaction |  |  |  |  |  |  | 0.03 | |  | 0.10 | |  |

BMI: body mass index; WC: waist circumference; s.d.: standard deviation; HR: hazard ratio. CI: confidence interval.

^1^ Models stratified by centre, age, and follow-up time, and adjusted for total energy intake, plausibility of energy reporting, alcohol intake, smoking status, educational level, recreational physical activity, and menopausal status (women). Body mass index models further adjusted by waist circumference; waist circumference models further adjusted by height and weight.

^2^ As 'whole cohort’ model plus further adjustment for daily intake of protein, carbohydrates, fiber, magnesium, red and processed meat, and coffee.

^3^ Exclusions: cancer (n = 304), angina (n = 387), acute myocardial infarction (n = 126), stroke (n = 163).

^4^ Change of diet due to gastro-intestinal problems, high blood pressure, hyperlipidemia, obesity, or other causes.

**Table S4. Hazard ratio (HR) and cumulative proportion of diabetes by combined BMI and WC categories in men and women from the Spanish EPIC (European Prospective Investigation into Cancer and Nutrition) cohort.**

|  |  | Py | Cases | HR^1^ | 95% CI |  |
| --- | --- | --- | --- | --- | --- | --- |
| **Men** |  |  |  |  |  |  |
| *BMI* | *WC* |  |  |  |  |  |
|  | < 94 cm | 19448 | 45 | 1 |  |  |
| < 25 kg/m^2^ | 94 - 101.9 cm | 4169 | 21 | 1.36 | 0.78 - 2.35 |  |
|  | ≥ 102 cm | 181 | 2 | 4.17 | 0.98 - 17.70 |  |
|  |  |  |  |  |  |  |
|  | < 94 cm | 26335 | 80 | 1.37 | 0.93 - 2.00 |  |
| 25 - 29.99 kg/m^2^ | 94 - 101.9 cm | 51955 | 314 | 1.98 | 1.43 - 2.73 |  |
|  | ≥ 102 cm | 21178 | 175 | 2.09 | 1.49 - 2.94 |  |
|  |  |  |  |  |  |  |
| ≥ 30 kg/m^2^ | < 94 cm | 607 | 7 | 2.58 | 1.12 - 5.96 |  |
|  | 94 - 101.9 cm | 7553 | 74 | 2.55 | 1.73 - 3.76 |  |
|  | ≥ 102 cm | 37652 | 554 | 2.79 | 2.02 - 3.85 |  |
| **Women** |  |  |  |  |  |  |
| *BMI* | *WC* |  |  |  |  |  |
|  | < 80 cm | 60892 | 32 | 1 |  |  |
| < 25 kg/m^2^ | 80 - 87.9 cm | 18898 | 22 | 1.76 | 1.01 - 3.06 |  |
|  | ≥ 88 cm | 3052 | 9 | 2.45 | 1.09 - 5.49 |  |
|  |  |  |  |  |  |  |
|  | < 80 cm | 20781 | 16 | 1.06 | 0.57 - 1.99 |  |
| 25 - 29.99 kg/m^2^ | 80 - 87.9 cm | 56710 | 130 | 2.47 | 1.64 - 3.72 |  |
|  | ≥ 88 cm | 45334 | 233 | 4.17 | 2.81 - 6.20 |  |
|  |  |  |  |  |  |  |
| ≥ 30 kg/m^2^ | < 80 cm | 563 | 0 | - |  |  |
|  | 80 - 87.9 cm | 6599 | 23 | 3.01 | 1.69 - 5.39 |  |
|  | ≥ 88 cm | 75060 | 776 | 5.60 | 3.80 - 8.25 |  |

BMI: body mass index. WC: waist circumference. Py: person-years. HR: hazard ratio. CI: confidence interval.

^1^ Models stratified by centre, age, and follow-up time, and adjusted for total energy intake, plausibility of energy reporting, alcohol intake, smoking status, educational level, recreational physical activity, and menopausal status (women).

**Table S5. Ten-years cumulative incidence (%) of diabetes (and 95% CI) according to waist circumference NCEP categories, stratified by body mass index, sex and age, in participants from the Spanish EPIC (European Prospective Investigation into Cancer and Nutrition) cohort.**

|  |  | Normal waist | | |  | Elevated waist^1^ | |
| --- | --- | --- | --- | --- | --- | --- | --- |
| Age at recruitment (years) |  | Normal waist | Overweight | Obese |  | Overweight | Obese |
| **Men** |  |  |  |  |  |  |  |
| 40 - 49 |  | 1.4 (0.7 - 2.1) | 2.9 (2.3 - 3.4) | 6.1 (3.7 - 8.4) |  | 4.7 (3.1 - 6.3) | 9.7 (8.1 - 11.3) |
| 50 - 59 |  | 3.0 (1.6 - 4.5) | 4.1 (3.2 - 4.9) | 9.0 (5.0 - 13.0) |  | 7.5 (5.6 - 9.4) | 13.5 (11.6 - 15.3) |
| 60 - 69 |  | 4.8 (1.9 - 7.7) | 7.0 (5.0 - 8.9) | 13.3 (4.6 - 22.0) |  | 11.7 (8.3 - 15.1) | 14.0 (11.2 - 16.8) |
| **Women** |  |  |  |  |  |  |  |
| 40 - 49 |  | 0.4 (0.2 - 0.7) | 1.1 (0.7 - 1.5) | 3.6 (1.3 - 5.9) |  | 2.8 (1.9 - 3.6) | 8.4 (7.2 - 9.5) |
| 50 - 59 |  | 0.9 (0.3 - 1.5) | 3.0 (2.1 - 3.8) | 3.2 (0.7 - 5.8) |  | 4.9 (3.8 - 6.0) | 9.3 (8.2 - 10.4) |
| 60 - 69 |  | 0.8 (0 -1.9) | 1.9 (0.7 - 3.1) | 5.0 (0 - 10.6) |  | 5.0 (3.3 - 6.7) | 10.6 (8.8 - 12.4) |

CI: confidence intervals; NCEP: National Cholesterol Education Program.

Elevated waist defined as a waist circumference equal to or higher than 102 cm in men or 88 cm in women.

Normal weight: body mass index (BMI) < 25 kg/m^2^; overweight: 25 kg/m^2^ ≤ BMI < 30 kg/m^2^; obese: BMI ≥ 30 kg/m^2^.

^1^ 'Normal weight' BMI category excluded because of low number of cases (n = 1 man, n = 6 women).

**Table S6. Population risk of diabetes attributable to excess body weight in men and women from the Spanish EPIC (European Prospective Investigation into Cancer and Nutrition) cohort.**

| Exposition | Prevalence of exposition among cases,  % (95% CI) | Relative  risk^1^  (95% CI) | Population attributable risk^2^,  % (95% CI) |
| --- | --- | --- | --- |
| Obesity |  |  |  |
| MEN | 49.9 (47.2 - 52.7) | 1.53 (1.35 - 1.73) | 17.3 (13.2 - 21.3) |
| WOMEN | 64.4 (61.7 - 67.0) | 1.94 (1.69 - 2.23) | 31.3 (26.7 - 35.8) |
| Overweight or obesity |  |  |  |
| MEN | 94.7 (93.4 - 95.9) | 1.94 (1.50 - 2.52) | 46.0 (33.4 - 58.6) |
| WOMEN | 94.9 (93.7 - 96.1) | 2.83 (2.14 - 3.74) | 61.4 (52.1 - 70.7) |

CI: confidence interval.

‘Obesity’ defined as body mass index (BMI) ≥ 30 kg/m^2^; ‘overweight or obesity’ defined as BMI ≥ 25 kg/m^2^.

^1^ Relative risk estimated as the hazard ratio derived from Cox regression models stratified by centre, age, and follow-up time, and adjusted by total energy intake, plausibility of energy reporting, alcohol intake, smoking status, educational level and recreational physical activity.

^2^ Attributable risk calculated as *p_d_ (RR - 1) / RR*; where *p_d_* = prevalence of exposition among cases, and *RR* = multivariable-adjusted hazard ratio of diabetes in exposed *versus* non-exposed participants.
